# Eye-tracking data and mathematical tasks with focus on mathematical reasoning

**DOI:** 10.1016/j.dib.2019.104216

**Published:** 2019-07-04

**Authors:** Mathias Norqvist, Bert Jonsson, Johan Lithner

**Affiliations:** a Department of Science and Mathematics Education, Umeå University, Umeå Mathematics Education Research Center, Sweden; b Department of Psychology, Umeå University, Sweden

**Keywords:** Eye tracking, Mathematics education, Mathematical reasoning, Problem solving

## Abstract

This data article contains eye-tracking data (i.e., dwell time and fixations), Z-transformed cognitive data (i.e., Raven's Advanced Progressive Matrices and Operation span), and practice and test scores from a study in mathematics education. This data is provided in a supplementary file. The method section describes the mathematics tasks used in the study. These mathematics tasks are of two kinds, with and without solution templates, to induce different types of mathematical reasoning.

**Table:** Specifications Table

Subject area	Mathematics Education
More specific subject area	Mathematical reasoning and problem solving
Type of data	Tables and figures.
How data was acquired	Data was gathered by eye-tracking using an EyeLink 1000.
Data format	Aggregated eye-tracking data in a supplementary Excel file.
Experimental factors	Participants were matched into two groups, based on cognitive proficiency, mathematics grade and gender.
Experimental features	Students solved mathematics tasks, with or without solution templates, while their eye-movements were recorded.
Data source location	Northern Sweden
Data accessibility	Eye-tracking data is supplied in a supplementary excel file. The mathematics tasks are presented as a table and with some figures in section 2 in this article.
Related research article	Norqvist, M. Jonsson, B. Lithner, J. Qwillbard, T., & Holm, L. Investigating algorithmic and creative reasoning strategies by eye tracking. *Journal of Mathematical Behavior*. https://doi.org/10.1016/j.jmathb.2019.03.008[1]

**Table:** 

**Value of the Data** • Eye-tracking is an objective method to observe student's task solving process, and can therefore be used to observe which information students utilize when solving tasks. This is crucial for understanding how task design can influence student's mathematical reasoning. • Eye-tracking data can be used to: ○ make additional analyses of students' eye movements. • Mathematics tasks can be used: ○ as a template to construct new tasks with the same reasoning requirements ○ to conduct replication studies.

## 1 Data

**Fig. 1:**
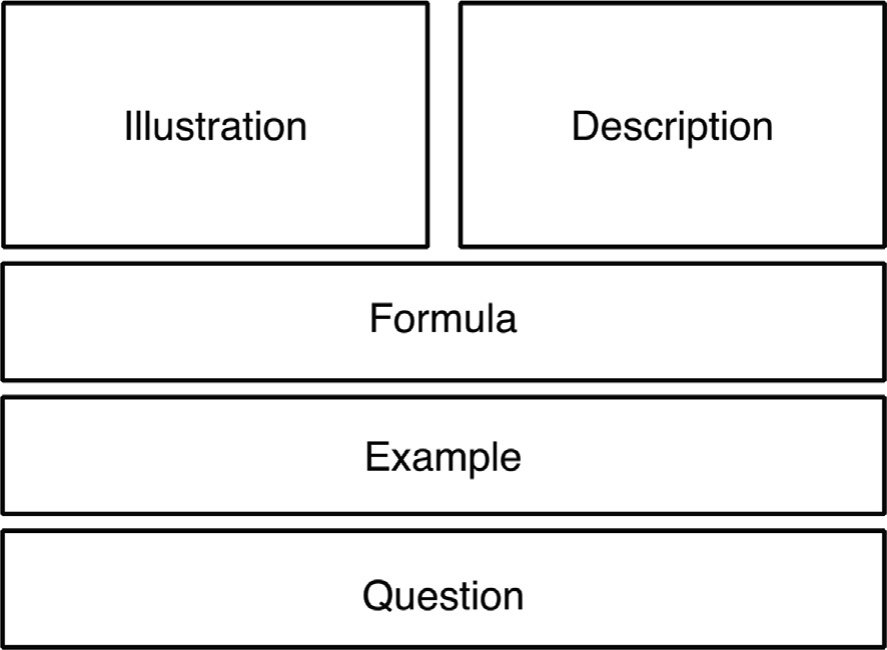
Task design with areas of interest (frames are not visible in the tasks presented to students).

**Fig. 2:**
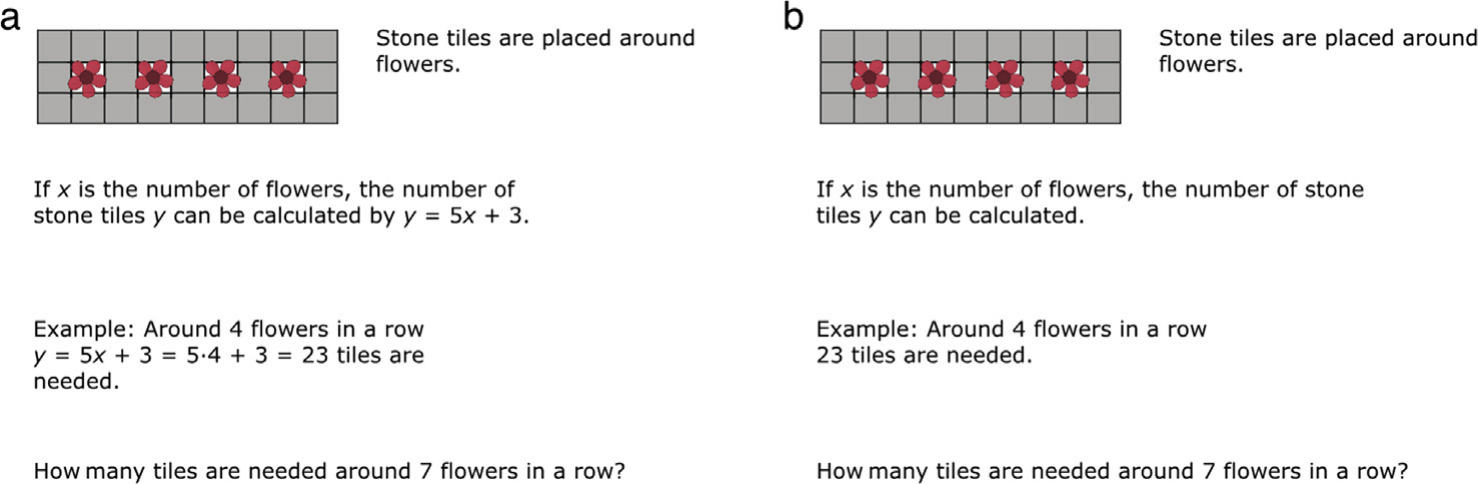
Example of the two task-types, with solution template (a) and without solution template (b).

While the participants worked with mathematics tasks described in section 2 (see Table 1, Table 2, Table 3 and Fig. 1, Fig. 2), eye-fixations and dwell time were recorded. Mean values of these variables as presented in the supplementary Excel file. Participant scores on Raven's Advanced Progressive Matrices and Operation Span are also included, as well as mathematical practice and test scores. The data file comprises two pages: the first contains the gathered data, as indicated above, and the second contains explanations of the abbreviated variables.

**Table 1:** Tasks with solution templates.

Task-set	Description	Formula	Example	Question
1	Squares are constructed with matches.	If *x* is the number of squares then the number of matches *y* can be calculated by *y* = 3*x* + 1.	Example: 4 squares can be made by *y* = 3*x* + 1 = 3·4 + 1 = 13 matches.	How many matches are needed for 6 squares?
2	Double-squares are constructed with matches	If *x* is the number of double-squares then the number of matches *y* can be calculated by *y* = 5*x* + 2.	Example: 4 squares can be made by *y* = 5*x* + 2 = 5·4 + 2 = 22 matches.	How many matches are needed for 7 squares?
3	Stone tiles are placed around flowers.	If *x* is the number of flowers, the number of stone tiles *y* can be calculated by *y* = 5*x* + 3.	Example: Around 4 flowers in a row *y* = 5*x* + 3 = 5·4 + 3 = 23 tiles are needed.	How many tiles are needed around 7 flowers in a row?
4	Stone tiles are placed around flower-triplets.	If *x* is the number of flower-triplets, the number of stone tiles *y* can be calculated by *y* = 11*x* + 7.	Example: Around 4 flower-triplets in a row *y* = 11*x* + 7 = 11·4 + 7 = 51 stone tiles are needed.	How many stone tiles are needed around 6 flower-triplets in a row?
5	Grey and yellow square tiles with a side length of 1 dm are mounted on a wall.	If the wall is *a* dm long and *b* dm high, the number of tiles *K* along the edges of the wall can be calculated by *K* = 2*a* + 2*b* −4.	Example: If the wall is 8 dm long and 6 dm high, *K* = 2·8 + 2·6–4 = 24 grey tiles are needed.	How many grey tiles are needed for the edge on a wall that is 9 dm · 7 dm?
6	Grey and white square tiles with a side length of 1 dm are mounted on a wall.	If the wall is *a* dm long and *b* dm high, the number of white tiles *A* can be calculated by A = *ab* -2*a* - 2*b* + 4.	Example: If the wall is 8 dm long and 6 dm high, *A* = 8·6–2·8 - 2·6 + 4 = 24 white tiles are needed.	How many white tiles are needed if the wall is 3 dm · 4 dm?
7	White square tiles with a side length of 3 dm is placed on a floor. Around the edge square grey tiles with side length 1 dm are placed.	If the rectangle with white tiles is *a* tiles long and *b* tiles wide, the number of grey tiles *R* can be calculated by *R* = 6*a* + 6*b* + 4.	Example: If the rectangle with white tiles is 3 tiles long and 2 tiles wide, *R* = 6·3 + 6·2 + 4 = 34 grey tiles are needed.	How many grey tiles are needed if the white rectangle is 3 tiles long and 4 tiles wide?
8	Matchstick houses are put together as a row house.	If *x* is the number of houses in a row house, the number of matches *y* can be calculated by *y* = 5*x* + 1.	Example: If the row house consists of 4 houses, *y* = 5·4 + 1 = 21 matches are needed.	How many matches are needed for a row house with 6 houses?
9	Matchstick houses are put together as a row house.	If *x* is the number of houses in a row house, the number of matches along the edge *y* can be calculated by *y* = 3*x* + 1.	Example: If the row house consists of 4 houses, *y* = 3·4 + 1 = 13 matches are needed for the edge.	How many matches are needed for the edge of a row house with 7 houses?
10	A quilt blanket is sewn out of light grey octagons, black squares, white, and dark grey triangles. The blanket has the shape of a square.	If the blanket contains *n·n* octagons, the number of dark grey triangles can be calculated by *T* = 4*n* – 4.	Example: If the blanket contains 3·3 octagons, *T* = 4·3–4 = 8 dark grey triangles are needed.	How many dark grey triangles are needed if the quilt blanket contains 5·5 octagons?

**Table 2:** Tasks without solution templates.

Task-set		Description	Formula	Example	Question
1		Squares are constructed with matches.	If *x* is the number of squares then the number of matches *y* can be calculated.	Example: 4 squares can be made by 13 matches.	How many matches are needed for 6 squares?
2		Double-squares are constructed with matches	If *x* is the number of double-squares then the number of matches *y* can be calculated.	Example: 4 squares can be made by 22 matches.	How many matches are needed for 7 squares?
3		Stone tiles are placed around flowers.	If *x* is the number of flowers, the number of stone tiles *y* can be calculated.	Example: Around 4 flowers in a row 23 tiles are needed.	How many tiles are needed around 7 flowers in a row?
4		Stone tiles are placed around flower-triplets.	If *x* is the number of flower-triplets, the number of stone tiles *y* can be calculated.	Example: Around 4 flower-triplets in a row 51 stone tiles are needed.	How many stone tiles are needed around 6 flower-triplets in a row?
5		Grey and yellow square tiles with a side length of 1 dm are mounted on a wall.	If the wall is *a* dm long and *b* dm high, the number of tiles *K* along the edges of the wall can be calculated.	Example: If the wall is 8 dm long and 6 dm high 24 grey tiles are needed.	How many grey tiles are needed for the edge on a wall that is 9 dm · 7 dm?
6		Grey and white square tiles with a side length of 1 dm are mounted on a wall.	If the wall is *a* dm long and *b* dm high, the number of white tiles *A* can be calculated.	Example: If the wall is 8 dm long and 6 dm high 24 white tiles are needed.	How many white tiles are needed if the wall is 3 dm · 4 dm?
7		White square tiles with a side length of 3 dm is placed on a floor. Around the edge square grey tiles with side length 1 dm are placed.	If the rectangle with white tiles is *a* tiles long and *b* tiles wide, the number of grey tiles *R* can be calculated.	Example: If the rectangle with white tiles is 3 tiles long and 2 tiles wide 34 grey tiles are needed.	How many grey tiles are needed if the white rectangle is 3 tiles long and 4 tiles wide?
8		Matchstick houses are put together as a row house.	If *x* is the number of houses in a row house, the number of matches *y* can be calculated.	Example: If the row house consists of 4 houses 21 matches are needed.	How many matches are needed for a row house with 6 houses?
9		Matchstick houses are put together as a row house.	If *x* is the number of houses in a row house, the number of matches along the edge *y* can be calculated.	Example: If the row house consists of 4 houses 13 matches are needed for the edge.	How many matches are needed for the edge of a row house with 7 houses?
10		A quilt blanket is sewn out of light grey octagons, black squares, white, and dark grey triangles. The blanket has the shape of a square.	If the blanket contains *n·n* octagons, the number of dark grey triangles can be calculated.	Example: If the blanket contains 3·3 octagons 8 dark grey triangles are needed.	How many dark grey triangles are needed if the quilt blanket contains 5·5 octagons?

**Table 3:** Illustrations for each Task-set.

Task-set	Illustration
1	
2	
3	
4	
5	
6	
7	
8	
9	
10

## 2 Experimental design, materials, and methods

The supplementary data set includes data from 48 upper secondary and university students. The students were matched into two similar groups based on cognitive scores, gender, and mathematics grade. Cognitive scores include Raven's Advanced Progressive Matrices [2] and Operation Span [3]. Raven's matrices is a standardized test of abstract non-verbal problem-solving while operation Span is a complex working-memory task which measures executive functions involved in coordinating the processing and storage of information (see Refs. [2], [3] for detailed information). The participants practiced on mathematics tasks while their eye-movements were recorded by an EyeLink 1000, video-based eye tracker with which was set to a sampling frequency of 500 Hz, a sampling duration above 50 ms and a gaze resolution of approximately one degree across participants (see Ref. [1] for more details). One week after the practice session, the students took a mathematics test to evaluate what they remembered from the practice session. For a detailed description of the method, see Ref. [1]. The data analysis was based on Block distance and Ward's method [4] which through an iterative process merged data into clusters of increasing dissimilarities (see supplementary data file and [4] for detailed information).

Two types of mathematics tasks are presented (Table 1, Table 2, Table 3). The general task design is indicated by Fig. 1, where the five areas of interest are marked. Examples can be seen in Fig. 2. The tasks in Table 1 contains a solution template, similar to textbooks, and were given to the first experiment group. The tasks in Table 2 do not include a solution template, and were given to the second experiment group. Table 3 contains the illustrations included the tasks. The difference between the two task types is the information given in the areas marked as ‘formula’ and ‘example’. The purpose of the tasks was to induce either imitative or creative mathematical reasoning [5]. Previous studies have shown that similar task designs fulfill this purpose [6], [7]. Within each task set additional tasks were given with different numbers in the question (e.g., How many matches are needed for 20 squares?).

## References

[1] Norqvist M., Jonsson B., Lithner J., Qwillbard T., Holm L. (2019). Investigating algorithmic and creative reasoning strategies by eye tracking. J. Math. Behav..

[2] Raven J. (1991). The raven progressive matrices: implications for fostering abilities. Eur. J. High Abil..

[3] Unsworth N., Heitz R.P., Schrock J.C., Engle R.W. (2005). An automated version of the operation span task. Behav. Res. Methods.

[4] Ward J.H. (1963). Hierarchical grouping to optimize an objective function. J. Am. Stat. Assoc..

[5] Lithner J. (2008). A research framework for creative and imitative reasoning. Educ. Stud. Math..

[6] Jonsson B., Norqvist M., Liljekvist Y., Lithner J. (2014). Learning mathematics through algorithmic and creative reasoning. J. Math. Behav..

[7] Norqvist M. (2018). The effect of explanations on mathematical reasoning tasks. Int. J. Math. Educ. Sci. Technol..

